# Book Notices

**Published:** 1897-03

**Authors:** 


### Book Notices.

THE PATHOLOGY AND SURGICAL TREATMENT OF TUMORS, by N. Senn, M. D., Ph. D., LL. D., Professor of Practice of Surgery and Clinical Surgery, Rush Medical College; Professor of Surgery, Chicago Polyclinic; Attending Surgeon to Presbyterian Hospital; Surgeon-in-Chief, St. Joseph's Hospital, Chicago. In one royal octavo volume of seven hundred and nine pages; illustrated by five hundred and fifteen engravings, including full page colored plates. Price, cloth, $6.00; half morocco, $7.00. For sale by subscription only. W. B. Saunders, Publishers, 925 Walnut Street, Philapelphia.

For the purpose of simplifying diagnosis the author endeavors to trace every tumor to its proper anatomical starting point and histogenetic source, and to make a sharp histological and clinical distinction between true tumors, inflammatory swellings, and retention cysts. His classification of tumors is based on the following theory of their origin. 1st. The increase in volume caused by a tumor is due entirely to erratic cell growth from a matrix of embryonal cells of congenital or post natal origin. 2nd. The enlargement of a part or an organ caused by chronic inflammation which so often simulates a tumor is due to proliferation of pre-existing mature cells acted upon by pathogenic micro-organisms on their toxines, and to the vascular changes and cell-migration characteristic of inflammation. 3rd. A retention-cyst essentially consists of an accumulation of physiological secretion in a pre-formed glandular space, the result of a mechanical obstruction.

The first part of this treatise is devoted to a general consideration of tumors. Following the section on Classification, each class of tumors is considered separately, beginning with benign epithelial tumors and terminating with sarcoma, to which is appended a section on Retention-cysts. A description of each class of tumors is followed by a consideration of the topographical distribution of that particular kind of tumor in the different regions and organs of the body, with a description of the different operative procedures for their removal.

The text is profusely illustrated throughout, the microscpical picture of the tumor, in many places, being contrasted with the normal structure of the tissues corresponding with the anatomical location of the tumor. The more difficult operations are also fully described and illustrated.

The value of a treatise such as this can be more fully appreciated when we consider the condensed space devoted to this branch of surgical pathology in our best text-books and systems of surgery. The profession is indebted to Dr. Senn for his painstaking labor in producing this most valuable work at this time, when it is so much needed. S. E. H.

---

THE AMERICAN YEAR-BOOK OF MEDICINE AND SURGERY, being a yearly digest of Scientific Progress and Authoritative Opinion in all Branches of Medicine and Surgery, drawn from Journals, Monographs and Text-Books of the leading American and Foreign Authors and Investigators, collected and arranged with critical editorial comments by eminent American Specialists and teachers. Under the editorial charge of George M. Gould, M. D. One volume of 1257 pages, profusely illustrated with numerous wood-cuts in text, and 20 full page plates. Price, cloth, $6.50; half Morrocco, $7.50. For sale by subscription. W. B. Saunders, publisher, 925 Walnut street, Philadelphia. 1897.

The cordial reception given by the profession to Saunders' 1896 Year-book encouraged him to continue the publication of this work. The present volume is some 75 pages larger than the 1896 issue, but remains at the same price. A few changes have been made in the departmental editors, but the book has suffered none by this change. The editors number 28, all of them eminent and earnest medical men, and this volume bears evidence that each one has done his full duty. The Year-book covers the whole domain of medicine and surgery, giving the latest knowledge on the various subjects and the real progress that has been made during the past year. One especially valuable feature of the 1897 Year-book is a brief recapitulation of the trend and results of the year's work preceding each article. The book is profusely illustrated. The mechanical work is excellent; in fact the publisher has spared no expense to make it one of the best in every respect. S. E. H.

---

TWENTIETH CENTURY PRACTICE.—An International Encyclopedia of Modern Medical Science. By Leading Authorities of Europe and America. Edited by Thomas L. Stedman, M. D., New York City. In Twenty Volumes. Volume X. "Diseases of the Nervous System." New York. William Wood & Company. 1897.

The opening article, on "Diseases of the Brain," is by Dr. Joseph Collins, of New York. This is an exhaustive treatise covering some 264 pages, and including a discussion of the morphology and anatomy of the brain, cerebral localization, acute hemorrhagic, purulent, and syphilitic encephalitis, superior and inferior, polioencephalitis and polioencephalomyelitis, diffused sclerosis of the brain, infantile cerebral palsies, multiple sclerosis, hydrocephalus, parasites of the brain, thrombosis of the dural sinuses, diseases of the cerebellum, diseases of the oblongata, chronic bulbar paralysis, primary vascular lesions of the oblongata, associated neuritis of the bulbar nerves, and asthenic bulbar paralysis. The second article is by Dr. Charles L. Dana, of New York, on "Intracranial Hemorrhage, Embolism, Thrombosis," and covers thirty-four pages. Dr. B. Sachs, of New York, contributes an article on "Tumors of the Brain," and Dr. Joseph Collins one on "Diseases of the Meninges." Dr. Charles S. Fere, of Paris, France, discusses "Hysteria," "Epilepsy" and the "Spasmodic Neuroses" in three most excellent articles. "Neurasthenia" is the subject of an article by Dr. Charles L. Dana. Dr. Howell T. Pershing, of Denver, discusses the "Disorders of Speech, and Dr. Sanger Brown, of Chicago, "The Disorders of Sleep." The volume contains 859 pages, and as a whole is a superior work on diseases of the nervous system. The publishers announce that Vol. IX. will follow in April. S. E. H.

